# Can the MMP-9/NGAL ratio be a diagnostic biomarker for the diagnosis of endometrioma in infertile patients?

**DOI:** 10.3389/fendo.2025.1624717

**Published:** 2025-07-30

**Authors:** Batuhan Turgay, Batuhan Özmen, Harun Kılıçkıran, Yavuz Emre Şükür, Murat Sönmezer, Bülent Berker, Cem Somer Atabekoğlu, Ruşen Aytaç

**Affiliations:** ^1^ Department of Obstetrics and Gynecology, Reproductive Endocrinology, School of Medicine, Ankara University, Ankara, Türkiye; ^2^ Hatay Dörtyol State Hospital Department of Obstetrics and Gynecology, Hatay, Türkiye

**Keywords:** MMP-9, NGAL, endometrioma, laparoscopy, infertility

## Abstract

**Objective:**

In this study, we aimed to investigate whether serum NGAL, MMP-9 and the MMP-9/NGAL ratio, which are inflammatory markers used for the diagnosis and follow-up of some diseases, can be used as diagnostic and follow-up markers for the diagnosis of endometriomas in infertile patients.

**Methods:**

Forty-five patients with unexplained infertility and 45 infertile patients with endometriomas were included in the study. Patients with endometriomas of at least 3 cm in size were included in the study. NGAL and MMP-9 levels in venous blood samples and the MMP-9/NGAL ratios of the unexplained infertility and endometrioma groups and the preoperative and postoperative results of the endometrioma group were compared.

**Results:**

The mean blood NGAL and MMP-9 levels in the endometrioma and unexplained groups were 22.0 ± 4.0 ng/ml and 25.4 ± 4.9 ng/ml and 43.7 ± 8.0 ng/ml and 39.3 ± 10.7 ng/ml, respectively, and all the results were statistically significant (p=0.001; p=0.012). The mean blood levels of NGAL and MMP-9 in endometriomas and the same patients at three months after surgery were 24.9 ± 4.9 ng/ml and 27.0 ± 4.9 ng/ml and 43.9 ± 7.3 ng/ml and 36.7 ± 8.7 ng/ml, respectively (p=0.179; p=0.006). The mean ratios of MMP-9/NGAL in the endometrioma, unexplained and postoperative groups were 2.0 ± 0.2, 1.5 ± 0.2 and 1.4 ± 0.2, respectively. All these results were significantly different between the endometrioma-unexplained group and the endometrioma-postoperative group (p=0.001; p=0.001). When we performed a ROC curve analysis for the presence of endometrioma, an MMP-9/NGAL ratio greater than 1.75 had 86.1% sensitivity and 84% specificity in indicating the presence of endometrioma (AUC=0.898). There was a positive correlation between the VAS score and the MMP-9/NGAL ratio.

**Conclusions:**

Interestingly, the NGAL blood NGAL level was lower in the endometrioma group than in the control group. The MMP-9/NGAL ratio can be useful in the diagnosis of endometrioma, and this ratio reflects the clinical findings of the disease.

## 1 Introduction

Endometriosis profoundly affects women’s reproductive health and is intricately linked to infertility. Research indicates that the existence of endometriomas substantially impacts women’s reproductive rates. Endometriosis may result in ovulatory dysfunction, diminished ovarian reserve, and fallopian tube obstructions. These diseases may diminish the likelihood of conception and contribute to infertility. Although endometriosis is a frequent and benign condition, dysmenorrhea, dyspareunia, chronic pain and infertility can also be related to endometriosis and the resulting inflammation (1). Chronic inflammation is a keystone of endometriosis and is characterized by the infiltration of immune cells, which promote an inflammatory microenvironment (2).

Inflammation is crucial in the pathophysiology of endometriosis and affects the local microenvironment of lesions and systemic immune responses. Endometriosis is often characterized by a chronic inflammatory state. The presence of ectopic endometrial tissue triggers an immune response that is typically abnormal and dysregulated. In contrast to individuals with the expected protective immune response, those with endometriosis frequently exhibit altered cytokine profiles and immune cell infiltration (3, 4). Additionally, a dysregulated immune response and altered inflammatory mediators can be obvious in endometriomas (5, 6).

NGAL and MMP-9 are acute-phase reactants. NGAL is linked to inflammation, whereas MMP-9 promotes destruction of the extracellular matrix, assisting in lesion invasion and adhesion (7). It is unclear whether serum NGAL levels change in the case of endometrioma, and while one of the two studies in the literature reported no difference, the other indicated that the NGAL level was higher in the endometrioma group (8, 9) On the other hand, recent articles have increasingly focused on the role of MMP-9 in reproductive health, particularly in the context of endometriosis and infertility. Endometriosis is associated with elevated levels of MMP-9 regardless of the biological compartment studied (10).

This study aimed to assess the efficacy of NGAL, MMP-9, and the MMP-9/NGAL ratio in diagnosing endometriomas. The data derived from the concurrent application of NGAL and MMP-9 may facilitate a novel method for diagnosing endometrioma and increase the understanding of the functions of these biomarkers in the disease mechanism. This work aims to expand the literature by identifying novel biomarkers for the diagnosis of endometrioma, a contributing factor to infertility.

## 2 Materials and methods

### 2.1 Study design

This prospective, case–control study included 90 infertile women who were divided into two groups: 45 women with a diagnosis of endometrioma (study group) and 45 women with unexplained infertility (control group). The study was conducted at Ankara University School of Medicine, Department of Obstetrics and Gynecology, Reproductive Endocrinology Clinic, and all participants provided written informed consent before enrollment. Detailed infertility investigations were conducted on the patients. The demographic features, hormone levels, ultrasound findings, and surgical reports of the patients who underwent surgery, as well as the findings from their postoperative follow-ups and preoperative-postoperative VAS scores on the first day of the menstrual cycle, were recorded. Ethical approval was obtained from the Ankara University School of Medicine Ethical Board (Number:İ11-656–22 Date:08.12.2022) and the study was conducted according to the Helsinki Declaration. The research was financed by Ankara University Scientific Research Projects (Number: TDK-2024-3596).

There are no other articles about this specific topic, so the size of the study population is determined according to the most similar article (8). There should be 35 patients in each group for a significant difference between the two groups, with a 5% margin of error and 80% power according to the G-power calculation. A total of 45 patients in each group were included because of the possibility of a decrease in the number of patients during the follow-up period.

The inclusion criteria are as follows:

- Age between 18–35 years. (for minimizing age-related variation in fertility and biomarker expression).
- Diagnosis of infertility.
- For the endometrioma group, the presence of endometrioma confirmed by ultrasonography, with a cyst diameter of at least 3 cm, and a definitive laparoscopic confirmation were performed. All of the patients are in the stage 3 according to the ASRM classification without dense adhesions or diffuse pelvic endometriosis.
- For the unexplained infertility group, infertility without identifiable causes after a full infertility examination, including tubal patency tests, ovulatory function assessment, and partner semen analysis within normal limits, was performed.
- Not taking NSAIDs or hormone medications for at least one month.

The exclusion criteria are as follows:

- Has any cause of infertility.
- Other pelvic inflammatory conditions, such as pelvic inflammatory disease or other ovarian cysts, are present.
- Diffuse peritoneal endometriosis.
- History of previous endometrioma surgery or other pelvic surgeries.
- Presence of systemic inflammatory conditions or autoimmune diseases.
- Use of hormonal medications or immunosuppressive agents within 3 months prior to sample collection.
- Any contraindications to laparoscopic surgery (for the endometrioma group).

There are no any consecutive cases and the control groups are matched for relevant factors.

### 2.2 Sample collection

Fasting venous blood samples (5 mL) were collected from each participant in the early follicular phase to reduce hormonal variability. For the endometrioma group, blood samples were collected twice: once preoperatively and once three months postoperatively.

Blood samples were drawn into sterile vacutainer tubes without anticoagulants and immediately transported to the laboratory. The samples were allowed to coagulate at ambient temperature for 30 minutes, followed by centrifugation at 3000 rpm for 10 minutes to isolate the serum. Serum was aliquoted into Eppendorf tubes and stored at -80°C until analysis to preserve the stability of NGAL and MMP-9 levels.

### 2.3 Measurement of biomarkers (NGAL and MMP-9)

Serum levels of NGAL and MMP-9 were assessed via enzyme-linked immunosorbent assay (ELISA) kits tailored for each biomarker. All ELISA techniques were executed in accordance with the written protocol, and all assays were run in duplicate to ensure precision (Shanghai Coon Koon Biotech Co., Ltd., Shanghai, China). The intra-assay and interassay coefficients for MMP-9 were less than 7% and less than 10%, respectively. The intra-assay and interassay coefficients for NGAL were less than 10% and less than 12%, respectively. Both kits’ sensitivity was 1.0 ng/mL. The MMP-9/NGAL ratio was determined by dividing the MMP-9 concentration by the NGAL concentration for each sample, functioning as an auxiliary biomarker for comparative analysis. All samples were processed uniformly and measurements were performed blindly.

### 2.4 Statistical analysis

Comparative statistical studies were performed to assess differences in NGAL and MMP-9 levels, together with the MMP-9/NGAL ratio, between the unexplained infertility and endometrioma cohorts. The preoperative and postoperative parameters for the endometrioma group were evaluated for changes after surgical intervention. p<0.05 was considered statistically significant. Receiver operating characteristic (ROC) curve analysis was conducted to evaluate the diagnostic efficacy of the MMP-9/NGAL ratio in differentiating endometriomas. The sensitivity, specificity, and area under the curve (AUC) values were examined to determine the optimal threshold for endometrioma detection.

## 3 Results

### 3.1 Demographic features

The average age in the endometrioma group was 31.53 (± 3.42) years, whereas in the unexplained infertility group, it was 30.28 (± 2.98) years (p=0.79). The mean BMI was 24.72 (± 1.79) for the endometrioma group and 23.82 (± 1.54) for the unexplained group (p=0.32). The average infertility duration was 2.82 (± 1.11) years for the endometrioma group and 2.55 (± 1.22) years for the unexplained group (p=0.33). Patients in the endometriosis group underwent surgery because of pain (n=24), a large adnexal mass (n=15) and difficulties in oocyte pick-up (n=6). (**Table 1**).

**Table 1:** Demographic features of the patients.

	Endometrioma (N:45)	Unexplained (N:45)	p
Age (years) (Mean ± SD)	32.53 ± 3.42	31.72 ± 4.98	0.79
BMI (kg/m^2^) (Mean ± SD)	24.72 ± 1.79	23.82 ± 1.54	0.32
Smoking(N)	3	2	0.43
Systemic disease(N)	5	6	0.72
Previous surgery(N)	10	8	0.65
Gravida (Mean ± SD)	1.21 ± 0.52	1.13 ± 0.41	0.51
Parity(Mean ± SD)	0.92 ± 0.33	0.88 ± 0.42	0.67
Abortus (Mean ± SD)	0.22 ± 0.21	0.45 ± 0.32	0.55
Infertility duration (years) (Mean ± SD)	2.82 ± 1.11	2.55 ± 1.22	0.33
Primary infertility (N)	21	24	0.12

The mean AMH levels were 1.89 (± 0.63) and 3.32 (± 2.31) in the endometrioma group and in the unexplained infertility group, respectively (p=0.04), indicating a statistically significant difference. The mean CA-125 level was 79.08 (± 48.95) in the endometrioma group (no data were available for the unexplained group). There was no statistically significant difference in the other parameters between these groups. (**Table 2**).

**Table 2:** Hormonal and blood parameters of patients.

	Endometrioma (N:45)	Unexplained (N:45)	p
AMH (ng/ml)	1.89 ± 0.63	3.32 ± 2.31	0.04
FSH (IU/L)	7.67 ± 2.21	6.93 ± 2.12	0.56
LH (mlU/ml)	4.21 ± 2.32	5.01 ± 2.89	0.41
E2 (pg/ml)	43.12 ± 10,92	39.58 ± 9.99	0.68
TSH (mU/ml)	2.98 ± 1.21	3.12 ± 1.05	0.77
Hemoglobin (g/dl)	11.94 ± 1.39	12.88 ± 1.32	0.91
White blood cell (mcL)	7.03 ± 2.33	7.42 ± 1.65	0.15
Neutrophil (mcL)	5.44 ± 6.85	4.66 ± 1.36	0.06
Lymphocyte (mcL)	2.02 ± 0.70	2.08 ± 0.56	0.25
CRP(mg/L)	4.02 ± 0.52	3.52 ± 0.58	0.18
CA-125 (U/ml)	79.08 ± 48.95	No data	

(Mean ± SD).

The mean antral follicle count was 10.52 (± 2.32) in the endometrioma group and 13.33 (± 2.11) in the unexplained infertility group (p=0.04), indicating a statistically significant difference. There were 6 cases of bilateral endometrioma in the endometrioma group, whereas no cases were reported in the unexplained infertility group. (**Table 3**).

**Table 3:** Ultrasonographic features of patients.

	Endometrioma (N:45)	Unexplained (N:45)	p
Antral follicle count (Mean ± SD)	10.52 ± 2.32	13.33 ± 2.11	0.04
Bilateral endometrioma (N)	6	No	
Endometrioma size (cm)	5.95 ± 2.39	No	

The mean NGAL level was 22.01 (± 4.04) ng/ml in the endometrioma group and 25.46 (± 4.91) ng/ml in the unexplained infertility group (p=0.001), indicating a statistically significant difference. The mean MMP-9 level was 43.74 (± 8.00) ng/ml in the endometrioma group and 39.38 (± 10.75) ng/ml in the unexplained infertility group (p=0.012). The mean MMP-9/NGAL ratio was 2.01 (± 0.24) in the endometrioma group and 1.51 (± 0.28) in the unexplained infertility group (p=0.001), indicating statistical significance. All the parameters are significantly different between the two groups. (**Table 4**).

**Table 4:** Biomarker levels of the endometrioma and unexplained groups.

	Endometrioma	Unexplained	p
NGAL(ng/ml)	22.01 ± 4.04	25.46 ± 4.91	0.001
MMP-9 (ng/ml)	43.74 ± 8.00	39.38 ± 10.75	0.012
MMP-9/NGAL	2.01 ± 0.24	1.51 ± 0.28	0.001

(Mean ± SD).

Four patients did not complete the follow-up, so their results were not included in the analysis when the postoperative outcomes were evaluated. The mean NGAL level was 24.9 (± 4.9) ng/ml preoperatively and 27.0 (± 4.98) ng/ml postoperatively, with no statistically significant difference (p=0.179). The mean MMP-9 level decreased from 43.9 (± 7.3) ng/ml preoperatively to 36.7 (± 8.7) ng/ml postoperatively, a statistically significant change (p=0.006). The MMP-9/NGAL ratio significantly decreased from 2.02 (± 0.23) preoperatively to 1.45 (± 0.26) postoperatively (p=0.001). VAS scores decreased significantly during the postoperative period (**Table 5**).

**Table 5:** Preoperative and postoperative NGAL, MMP-9, and CA-125 levels and VAS scores of the endometrioma group.

	Preoperative	Postoperative	p
NGAL(ng/ml)	24.9 ± 4.9	27.0 ± 4.98	0.179
MMP-9 (ng/ml)	43.9 ± 7.3	36.7 ± 8.7	0.006
MMP-9/NGAL	2.02 ± 0.23	1.45 ± 0.26	0.001
CA-125(U/ml)	77.06 ± 46.35	68.89 ± 49.27	0.146
VAS score	7.1 ± 2.1	4.9 ± 1.2	0.04

(Mean ± SD).

A regression analysis is being conducted to investigate whether the presence of endometrioma is related to age, BMI, AMH, NGAL, MMP-9, MMP-9/NGAL levels. The coefficient for the MMP-9/NGAL ratio is 5.176, with a standard error of 1.322 and a high odds ratio of 176.93, which is statistically significant (p=0.001) (**Table 6**).

**Table 6:** The regression analysis of the biomarker in the presence of endometrioma.

	B	S.E.	Exp(B)	p
Constant	-13.022	3.454	–	0.001
NGAL	-0.153	0.078	0.858	0.065
MMP-9	0.053	0.036	1.054	0.145
MMP-9/NGAL	5.176	1.322	176.93	0.001
AMH	-0.021	0.017	0.979	0.250
Age	-0.012	0.010	0.988	0.300
BMI	0.008	0.012	1.008	0.420

The MMP-9/NGAL ratio was strongly positively correlated with the presence of endometrioma (R = 0.691, p=0.01) (**Table 7**). The VAS score significantly correlated with the MMP-9/NGAL level (R= 0.712, p=0.01). The MMP-9/NGAL ratio was negatively correlated with the AMH value (R= -0.282, p=0.01).

**Table 7:** Correlations between the presence of endometrioma and NGAL, MMP-9, and the MMP-9/NGAL ratio.

	NGAL	MMP-9	MMP-9/NGAL
Presence of endometrioma	-0.361^*^	0.228^*^	0.691^**^

**p=0.01 ^**^ p=0.05.

**Figure 1:**
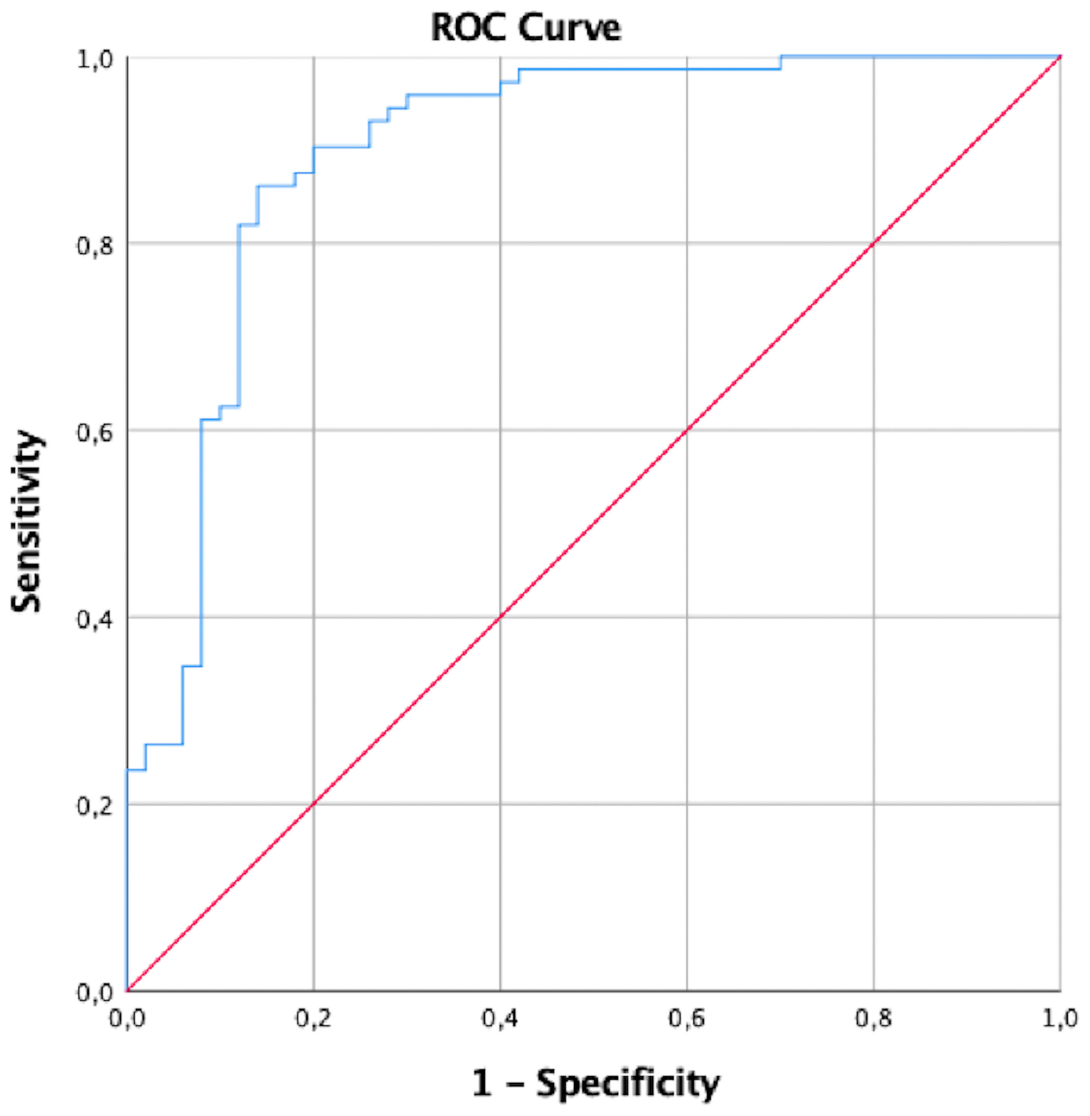
ROC analysis of the MMP-9/NGAL ratio in endometriomas.

ROC analysis was conducted to determine the ability of the MMP-9/NGAL ratio to predict the presence of endometrioma (**Figure 1**).

The cutoff value is determined by Youden index. This table summarizes the diagnostic performance of a cutoff value of 1.75 for the MMP-9/NGAL ratio. The sensitivity of these cutoff values was 86.1%, and the specificity was 84% (**Table 8**).

**Table 8:** The cutoff value of the MMP-9/NGAL ratio for the presence of endometrioma.

Cutoff	AUC(95%)	Sensitivity(95% CI)	Specificity(95% CI)	p	Lower bound	Upper bound
1.75	0.898 (0.837–0.960)	86.1% (77.9–91.5%)	84% (75.6–89.9%)	0.001	0.837	0.960

## 4 Discussion

This study investigated the serum levels of NGAL and MMP-9 in women with endometrioma and unexplained infertility, adding to the growing body of research identifying these markers as key contributors to endometriosis. Our findings revealed significantly elevated MMP-9 levels and reduced NGAL levels in patients with endometrioma compared with those with unexplained infertility, reinforcing the hypothesis that these biomarkers may play essential roles in the pathophysiology and potential diagnosis of endometriosis. According to our initial hypothesis, we expected the MMP-9 level to be high in the endometrioma group, and the results we obtained were consistent with this finding. However, surprisingly, a decrease in NGAL levels was observed in the endometrioma group compared with the unexplained infertility group. According to our initial hypothesis, this result was different. However, an MMP-9/NGAL ratio above 1.75 indicates the presence of endometrioma, with 86.1% sensitivity and 84% specificity. There was a positive correlation between the VAS score and the MMP-9/NGAL ratio.

NGAL is a lipocalin protein secreted by neutrophils in response to infection and inflammation, and NGAL is recognized to be elevated in instances of tissue injury (11). NGAL levels have been demonstrated to be applicable for the diagnosis and prognosis of many disorders characterized by significant inflammation (12). Importantly, the NGAL levels were low in the endometrioma cohort in our investigation. The literature indicates that NGAL levels are elevated in inflammatory processes such as endometriosis; however, its reduced levels in endometriomas imply that the microenvironment of endometriotic tissue may possess distinct properties. This microenvironment may possess a distinctive structure that inhibits NGAL production or diminishes circulating NGAL levels. The inhibition of NGAL expression may be caused by the local immunological milieu, which includes a changed ratio of anti-inflammatory cytokines including TGF-β and IL-10. Both TGF-β and IL-10 are well-known immunoregulatory cytokines that can prevent neutrophil activation and reduce the production of pro-inflammatory genes (13). These cytokines may directly or indirectly decrease NGAL transcription by downregulating pro-inflammatory transcription factors (NF-κB, for example) or encouraging regulatory T cell activity. This implies that the lower NGAL levels may be explained by local immunosuppressive circumstances, which are frequently seen in chronic inflammatory or tumor microenvironments. Consequently, a more precise assessment of NGAL expression patterns in diseased tissues requires taking into account the local cytokine milieu. Since the patients in our study did not have diffuse peritoneal endometriosis, there may not be venous NGAL elevation also. In a study conducted in Türkiye in 2019 on this topic, the urinary NGAL results of 60 endometriosis patients and 30 control patients were examined, and no difference was found between the groups. In the same study, when patients were grouped according to whether endometriosis was mild or severe, no significant difference was found (8). In another study conducted in 2023, 36 patients with endometriomas and 36 control patients were included, and the amount of NGAL was reported to be significantly greater in patients with endometriomas (9). To our knowledge, there are no other studies in the literature regarding endometrioma and NGAL levels, and these three studies, along with ours, have shown that NGAL levels do not change, decrease, or increase with the presence of endometrioma. These differences in NGAL levels revealed in the studies may be due to the differences in body fluids in which the material is studied, the way the kit is studied, the age, hormone profile, inflammation status of the patients and the severity of endometriosis. Therefore, the exact relationship between the NGAL level and the presence of endometrioma cannot be determined.

MMP-9 is involved in the remodeling of the extracellular matrix through proteolytic activity. It plays a key role in physiological and pathophysiological uterine processes (14). Elevated MMP-9 levels in endometriotic lesions may facilitate ectopic endometrial tissue growth, thereby promoting disease progression and symptom severity. In our study, the serum MMP-9 level was significantly greater in the endometrioma group than in the unexplained group. According to a review conducted in 2024, four candidate biomarkers should be considered in the context of endometriosis. These biomarkers are TNF-α, MMP-9, TIMP−1 and miR451, which are supported by at least two distinct research teams and are located in at least three or more biological compartments (15). This review indicates that MMP-9 levels are consistently elevated in endometriosis across all investigations and irrespective of the biological compartment examined. Another meta-analysis including 15 papers with 996 endometriosis patients and 582 non endometriosis patients reported that the concentration of MMP-9 in patients with endometriosis was markedly elevated compared with that in the control group (10). In this review, they also performed a subgroup analysis because the articles included in this meta-analysis included various study populations, such as uterine fibroids, uterine polyps and other diseases. Even if the patients had other gynecological pathologies, MMP-9 levels were still higher in the endometriosis group than in the control group. Additionally, the severity of the disease was reported to be positively correlated with the MMP-9 level.

A study excluded from the aforementioned meta-analysis demonstrated a significant increase in blood MMP-9 levels in the endometriosis group (8). The diagnostic efficacy of this biomarker seems appropriate, although it remains unverified. Our findings regarding elevated MMP-9 levels in endometriomas align with existing research (8). Establishing a cutoff value for this issue is necessary; however, the usage of various kits that produce results in different units complicates the matter. A 2013 study indicated that an increase in MMP-9 was related to inadequate oocyte and embryo development in women with endometriosis undergoing IVF. The equilibrium of MMP-9/TIMP-1 in these women was markedly disturbed, and the injection of progesterone appeared to dramatically rectify this imbalance, hence indirectly increasing the IVF success rate (16).

The primary finding of this investigation was the diagnostic efficacy of the MMP-9/NGAL ratio, which demonstrated an AUC of 0.898, with sensitivity and specificity values of 86.1% and 84%, respectively. The increased diagnostic accuracy suggests that it may serve as a crucial noninvasive biomarker for endometrioma. To our knowledge, the examination of this ratio within the endometriosis cohort was performed in a single study. This study revealed a significant increase in the MMP-9/NGAL ratio in the endometriosis cohort compared with the control group and in the stage III/IV endometriosis group compared with the stage I/II group. Research has shown that conventional markers such as CA-125 have limited effectiveness, as they often lack the specificity necessary to differentiate endometriosis from other gynecological conditions (17). The results of the current study validate the diagnostic significance of the MMP-9/NGAL ratio, suggesting that it may exceed CA-125 and other markers by offering a more direct evaluation of extracellular matrix remodeling and inflammatory processes associated with endometriosis. The positive significant correlation between the VAS score and the MMP-9/NGAL ratio is clinically significant. Accordingly, the systemic inflammatory response in patients may be the most important cause of pain and this ratio can be used to measure pain in the future instead of the subjective VAS test. The specificity of CA 125≥30u/ml was 96% (95% CI 81.7–99.9%) and sensitivity was 57% (95% CI 37.4–74.5%).

In line with recent reviews, biomarkers that directly reflect ECM degradation and inflammation may provide better diagnostic insights than general inflammatory markers alone. NGAL and MMP-9 not only are reflective of the inflammatory milieu in endometriotic lesions but are also are related to disease severity and invasiveness. Studies suggest that in advanced stages of endometriosis, the levels of these biomarkers tend to increase in serum and peritoneal fluid, which is correlated with the extent of endometrial invasion and inflammatory activity.

The biomarkers used for follow-up in chronic diseases are very important for clinical practice. CA-125 is usually used for the diagnosis and follow-up of endometriosis, but a Cochrane meta-analysis revealed that CA-125 and other biomarkers cannot meet the criteria for use as diagnostic tools (17). Additionally, a previous study reported that VEGF is a more sensitive follow-up biomarker than CA-125 is (18). The use of CA 125 ≥ 30 units/ml as a diagnostic biomarker for the endometriosis; pooled specificity was 96% (95% CI 81.7–99.9%) and sensitivity was 57% (95% CI 37.4–74.5%) (19). CA 125 shown significantly greater sensitivity for diagnosing moderate or severe endometriosis in comparison to minimal disease. CA-125 value is affected by many diseases, so its sensitivity is relatively low compared to the MMP-9/NGAL ratio. On the other hand, MMP-9/NGAL ratio has a higher specific issue for the inflammatory process and has a greater cycle-phase stability. Our study revealed that the MMP-9 level and the MMP-9/NGAL ratio decreased significantly during the postoperative period, but the NGAL and CA-125 levels did not change significantly. Therefore, the MMP-9 level and ratio can be used for the follow-up period of endometriomas, especially if the MMP-9 level is checked preoperatively. With surgical intervention, the inflammatory process is resolved by excision of the tissue causing inflammation and the MMP-9/NGAL ratio decreases. This may indicate that the disease is regressing.

In the current study, AMH value was found to be significantly lower in the endometrioma group. Recent meta-analyses and systematic reviews have increasingly highlighted the complex relationship between anti-Müllerian hormone (AMH) levels and endometriosis, emphasizing the impact of chronic inflammation on ovarian reserve. Several studies have demonstrated that women with endometriosis tend to have significantly lower serum AMH levels compared to controls, particularly in advanced stages of the disease or following surgical intervention (20). This decline in AMH is thought to reflect both the local inflammatory milieu and oxidative stress within the ovarian cortex, which can accelerate follicular atresia and disrupt granulosa cell function (21). In our results, a significant negative correlation was found between AMH and MMP-9/NGAL ratio and this result shows that inflammatory processes are effective in the decrease of AMH.

The strengths of this study are its exclusive focus on patients with infertility issues and the exclusion of those with other diseases that could cause inflammation. This study offers significant insights; however, it has limitations. The sample size, while adequate for statistical analysis, constrains the generalizability of the results. There may also be patients with subclinical endometriosis in the unexplained infertility group. Furthermore, our research focused exclusively on women with endometrioma or idiopathic infertility, omitting other stages of endometriosis that can have unique biomarker profiles. Subsequent research should focus on including larger and more diverse patient cohorts to assess the MMP-9/NGAL ratio throughout various phases of endometriosis. Additionally, further studies are needed to evaluate the temporal variations in NGAL and MMP-9 levels in relation to different therapeutic interventions, potentially enhancing their effectiveness in tracking illness development and recurrence.

Considering the constraints of existing diagnostic instruments, it presents a viable noninvasive option that could enhance early diagnosis and patient outcomes. Ongoing research is elucidating the molecular processes of NGAL and MMP-9 interactions in endometriosis, potentially facilitating their integration into individualized treatment regimens and disease monitoring, thereby enhancing endometriosis management. MMP-9, especially the MMP-9/NGAL ratio, can be used as a diagnostic and follow-up biomarker in endometriosis. For the use of these markers in the diagnosis of endometriosis, further studies are needed. In future studies, markers such as CA-125 can be added along with these biomarkers to create a nomogram that can show the severity of the disease.

## Data Availability

The raw data supporting the conclusions of this article will be made available by the authors, without undue reservation.
